# Diaqua­bis(2,5-di-4-pyridyl-1,3,4-thia­diazole-κ*N* ^2^)bis­(thio­cyanato-κ*N*)copper(II) dihydrate

**DOI:** 10.1107/S1600536808008854

**Published:** 2008-04-10

**Authors:** Wei-Wu Ma, Ming-Hua Yang

**Affiliations:** a Department of Chemistry, Lishui University, 323000 Lishui, ZheJiang, People’s Republic of China

## Abstract

In the title compound, [Cu(NCS)_2_(C_12_H_8_N_4_S)_2_(H_2_O)_2_]·2H_2_O, the Cu atom is located on an inversion center and displays an octa­hedral geometry. Two N atoms of two different 2,5-di-4-pyridyl-1,3,4-thia­diazole ligands and two N atoms from two separate thio­cyanate mol­ecules form the equatorial plane, while two coordinated water mol­ecules are in axial positions. The crystal structure is consolidated by extensive hydrogen bonding, forming a two-dimensional network.

## Related literature

For related literature, see: Moulton & Zaworotko (2001 ▶); Su *et al.* (2003 ▶); Zhang *et al.* (2005 ▶); Zhou *et al.* (2006 ▶).
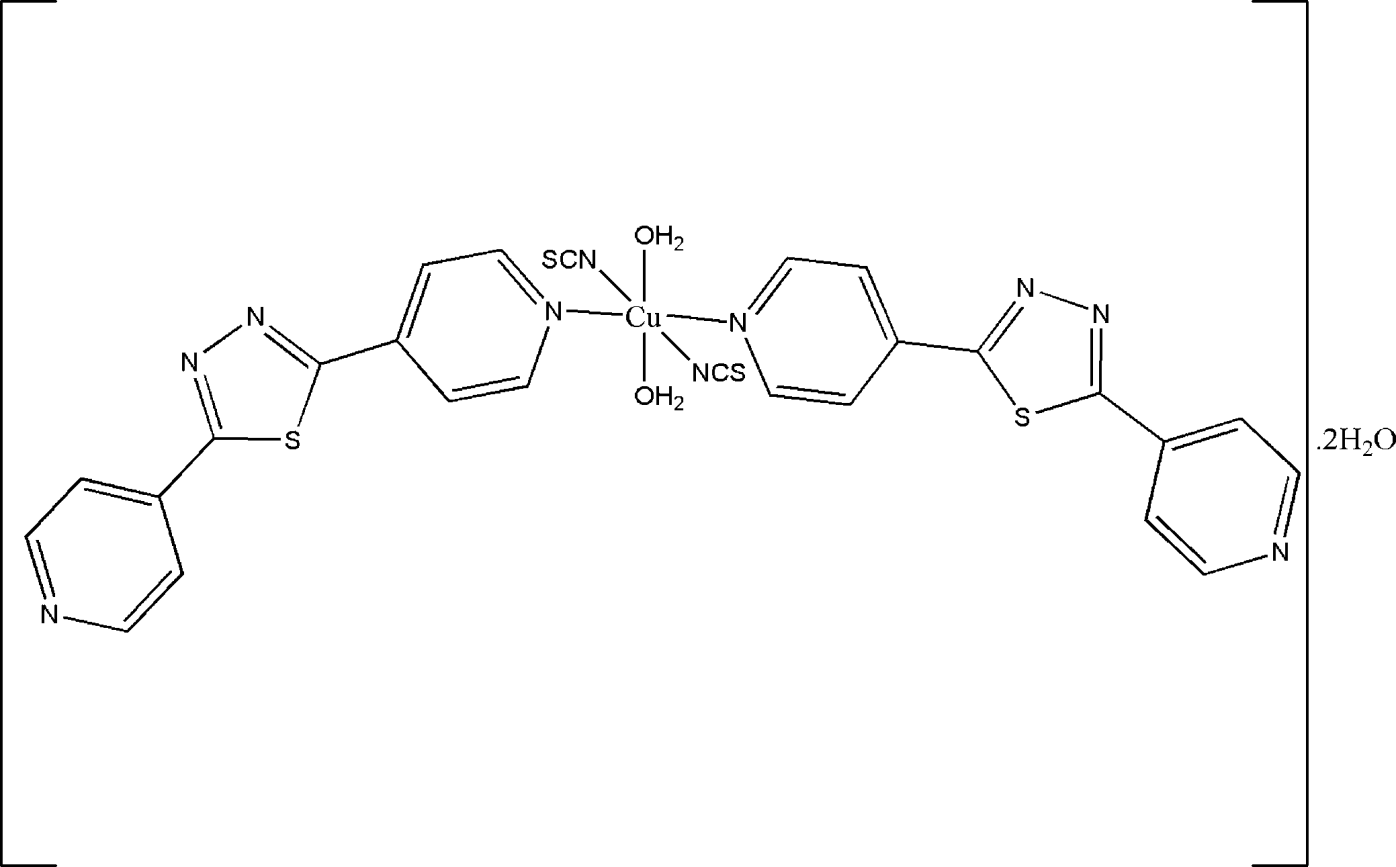


## Experimental

### 

#### Crystal data

[Cu(NCS)_2_(C_12_H_8_N_4_S)_2_(H_2_O)_2_]·2H_2_O
*M* *_r_* = 732.33
Triclinic, 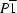

*a* = 7.0555 (11) Å
*b* = 8.3034 (13) Å
*c* = 14.849 (2) Å
α = 104.629 (2)°
β = 93.067 (2)°
γ = 112.228 (2)°
*V* = 768.3 (2) Å^3^
*Z* = 1
Mo *K*α radiation
μ = 1.03 mm^−1^
*T* = 298 (2) K
0.28 × 0.24 × 0.19 mm

#### Data collection

Bruker APEXII area-detector diffractometer
Absorption correction: multi-scan (*SADABS*; Sheldrick, 2004 ▶) *T*_min_ = 0.760, *T*_max_ = 0.828
3905 measured reflections
2692 independent reflections
1794 reflections with *I* > 2σ(*I*)
*R*_int_ = 0.028

#### Refinement

*R*[*F* ^2^ > 2σ(*F* ^2^)] = 0.061
*wR*(*F* ^2^) = 0.169
*S* = 1.07
2692 reflections
205 parameters
H-atom parameters constrained
Δρ_max_ = 0.44 e Å^−3^
Δρ_min_ = −0.69 e Å^−3^

### 

Data collection: *APEX2* (Bruker, 2004 ▶); cell refinement: *SAINT* (Bruker, 2004 ▶); data reduction: *SAINT*; program(s) used to solve structure: *SHELXS97* (Sheldrick, 2008 ▶); program(s) used to refine structure: *SHELXL97* (Sheldrick, 2008 ▶); molecular graphics: *ORTEPIII* (Burnett & Johnson, 1996 ▶), *ORTEP-3 for Windows* (Farrugia, 1997 ▶) and *PLATON* (Spek, 2003 ▶); software used to prepare material for publication: *SHELXL97*.

## Supplementary Material

Crystal structure: contains datablocks I, global. DOI: 10.1107/S1600536808008854/dn2330sup1.cif

Structure factors: contains datablocks I. DOI: 10.1107/S1600536808008854/dn2330Isup2.hkl

Additional supplementary materials:  crystallographic information; 3D view; checkCIF report

## Figures and Tables

**Table 1:** Hydrogen-bond geometry (Å, °)

*D*—H⋯*A*	*D*—H	H⋯*A*	*D*⋯*A*	*D*—H⋯*A*
O2—H2*B*⋯N5	0.82	2.03	2.835 (6)	169
O2—H2*C*⋯S2^i^	0.82	2.90	3.541 (4)	137
O1—H1*B*⋯S2^ii^	0.82	2.50	3.303 (4)	164
O1—H1*C*⋯O2^iii^	0.82	1.95	2.761 (6)	171

Symmetry codes: (i) 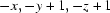; (ii) 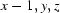; (iii) 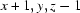.

**Fig. 1.**
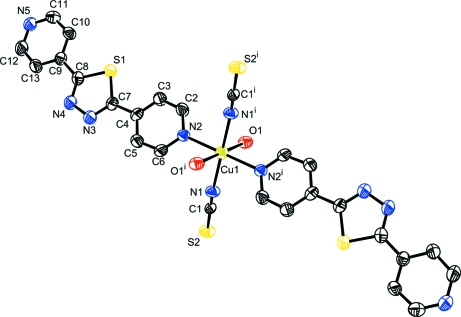
Molecular view of (I), with the atom labelling scheme. Displacement ellipsoids are drawn at the 30% probability level. Water molecule and H atoms have been omitted for clarity. [Symmetry code: (i) 1 - x, 2 - y, -z].

**Fig. 2.**
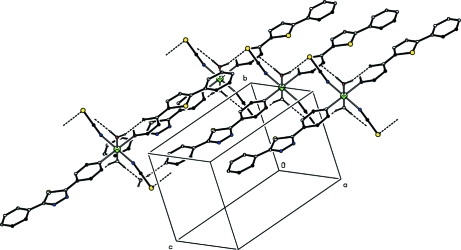
Partial packing view of (I), showing O—H···O, O—H···S and O—H···N hydrogen bonds leading to the formation of two-dimensional network. Hydrogen bonds are shown as dashed lines.

## supplementary crystallographic information

### Comment

In recent years, the rational design and assembly of metal-organic frameworks (MOFs) with well regulated network structures have received remarkable attention in order to develop new functional materials with potential applications (Moulton & Zaworotko, 2001). Nevertheless, it is still a great challenge to predict the exact structures and compositions of polymeric compounds assembled in a motifs, although some structures with various architectures have been reported in MOFs. So far, much of the research has been concentrated on the exploitation of angular ligands with a molecular angle, such as ligands with a T-shape, V-shape *etc*, in the construction of versatile coordination polymer architectures (Su *et al.*, 2003, Zhou *et al.*, 2006). However, the bent 2,5-di-4-pyridyl-1,3,4-thiadiazole (*L*), have been less studied as building blocks in the construction of metal-organic frameworks (Zhang *et al.*; 2005). The angular 2,5-di-4-pyridyl-1,3,4-thiadiazole has flexible coodination modes than general 4,4'-bipyrdine-like ligands due to two more potential N-donors atoms. In this paper, we report the synthesis and crystal structure of the title complex with a multifunctional *L* ligand,(I).

The Cu atom is located on an inversion center and displays  octahedral geometry (Fig. 1). Two nitrogen atoms of two different 2,5-di-4-pyridyl-1,3,4-thiadiazole ligands and two nitrogen atoms from two separated thiocyanate molecules form the basal plane, while two coordinated water molecules hold in axis position. The bond and angle are similar with others complexes with *L* ligand (Zhang *et al.*, 2005). These monuclear units are held together by means of H bonds involving the coordinated water molecules, sulfur atoms of thiocyanate, lattice water molecules and N atoms of pyridyl rings from *L* ligands, which further assemble into a 2-D supramolecular sheet (Fig.2, Table 1).

### Experimental

Cu(NCS)_2_(0.025 g, 0.13 mmol), *L*(0.031 g, 0.21 mmol), and NaOH (0.08 g, 0.2 mmol). were added in a solvent of methanol, the mixture was heated for ten hours under reflux. During the process stirring and influx were required. The resultant was then filtered to give a pure solution which was infiltrated by diethyl ether freely in a closed vessel, Four weeks later some single crystals of the size suitable for X-Ray diffraction analysis.

### Refinement

All H atoms attached to C atoms  were fixed geometrically and treated as riding with C—H = 0.93 Å (methyl) and U_iso_(H) = 1.2U_eq_(C or N). H atoms of water molecule were located in difference Fourier maps and included in the subsequent refinement using restraints (O-H= 0.82 (1)Å and H···H= 1.38 (2)Å) with U_iso_(H) = 1.5U_eq_(O). In the last stage of refinement they were treated as riding on their parent O atoms.

### Crystal data

**Table:**

[Cu(NCS)_2_(C_12_H_8_N_4_S)_2_(H_2_O)_2_]·2H_2_O	*Z* = 1
*M**_r_* = 732.33	*F*_000_ = 375
Triclinic, *P*1	*D*_x_ = 1.583 Mg m^−^^3^
Hall symbol: -P 1	Mo *K*α radiation λ = 0.71073 Å
*a* = 7.0555 (11) Å	Cell parameters from 2692 reflections
*b* = 8.3034 (13) Å	θ = 1.4–25.1º
*c* = 14.849 (2) Å	µ = 1.03 mm^−^^1^
α = 104.629 (2)º	*T* = 298 (2) K
β = 93.067 (2)º	Block, blue
γ = 112.228 (2)º	0.28 × 0.24 × 0.19 mm
*V* = 768.3 (2) Å^3^	

### Data collection

**Table:**

Bruker APEXII area-detector diffractometer	2692 independent reflections
Radiation source: fine-focus sealed tube	1794 reflections with *I* > 2σ(*I*)
Monochromator: graphite	*R*_int_ = 0.028
*T* = 298(2) K	θ_max_ = 25.1º
φ and ω scans	θ_min_ = 1.4º
Absorption correction: multi-scan(SADABS; Sheldrick, 2004)	*h* = −8→5
*T*_min_ = 0.761, *T*_max_ = 0.828	*k* = −9→9
3905 measured reflections	*l* = −17→17

### Refinement

**Table:**

Refinement on *F*^2^	Secondary atom site location: difference Fourier map
Least-squares matrix: full	Hydrogen site location: inferred from neighbouring sites
*R*[*F*^2^ > 2σ(*F*^2^)] = 0.061	H-atom parameters constrained
*wR*(*F*^2^) = 0.169	*w* = 1/[σ^2^(*F*_o_^2^) + (0.0774*P*)^2^ + 0.6059*P*] where *P* = (*F*_o_^2^ + 2*F*_c_^2^)/3
*S* = 1.07	(Δ/σ)_max_ < 0.001
2692 reflections	Δρ_max_ = 0.44 e Å^−^^3^
205 parameters	Δρ_min_ = −0.69 e Å^−^^3^
Primary atom site location: structure-invariant direct methods	Extinction correction: none

### Special details

**Table:**

Geometry. All esds (except the esd in the dihedral angle between two l.s. planes) are estimated using the full covariance matrix. The cell esds are taken into account individually in the estimation of esds in distances, angles and torsion angles; correlations between esds in cell parameters are only used when they are defined by crystal symmetry. An approximate (isotropic) treatment of cell esds is used for estimating esds involving l.s. planes.
Refinement. Refinement of *F*^2^ against ALL reflections. The weighted *R*-factor *wR* and goodness of fit *S* are based on *F*^2^, conventional *R*-factors *R* are based on *F*, with *F* set to zero for negative *F*^2^. The threshold expression of *F*^2^ > σ(*F*^2^) is used only for calculating *R*-factors(gt) *etc*. and is not relevant to the choice of reflections for refinement. *R*-factors based on *F*^2^ are statistically about twice as large as those based on *F*, and *R*- factors based on ALL data will be even larger.

### Fractional atomic coordinates and isotropic or equivalent isotropic displacement parameters (Å^2^)

**Table:**

	*x*	*y*	*z*	*U*_iso_*/*U*_eq_	
Cu1	0.5000	1.0000	0.0000	0.0423 (3)	
S1	−0.0367 (2)	0.7797 (2)	0.40422 (10)	0.0484 (4)	
S2	0.7893 (2)	0.5558 (2)	−0.03644 (12)	0.0477 (4)	
N1	0.6831 (7)	0.8539 (6)	−0.0062 (3)	0.0417 (11)	
N2	0.4011 (6)	0.9385 (6)	0.1283 (3)	0.0349 (10)	
N3	0.3261 (7)	0.7924 (7)	0.4413 (3)	0.0486 (13)	
N4	0.2304 (8)	0.7563 (7)	0.5165 (3)	0.0489 (13)	
N5	−0.3536 (8)	0.6276 (7)	0.7101 (3)	0.0511 (13)	
O1	0.2491 (5)	0.7683 (5)	−0.0897 (3)	0.0434 (9)	
H1B	0.1349	0.7366	−0.0728	0.065*	
H1C	0.2596	0.6773	−0.1223	0.065*	
O2	−0.6718 (6)	0.4775 (6)	0.8112 (3)	0.0586 (12)	
H2B	−0.5687	0.5204	0.7880	0.088*	
H2C	−0.6415	0.4525	0.8582	0.088*	
C1	0.7271 (8)	0.7310 (8)	−0.0188 (4)	0.0347 (12)	
C2	0.2117 (9)	0.9123 (8)	0.1465 (4)	0.0436 (14)	
H2	0.1223	0.9275	0.1043	0.052*	
C3	0.1394 (9)	0.8639 (8)	0.2240 (4)	0.0455 (14)	
H3	0.0025	0.8407	0.2316	0.055*	
C4	0.2713 (8)	0.8505 (7)	0.2895 (4)	0.0379 (13)	
C5	0.4715 (9)	0.8795 (8)	0.2725 (4)	0.0475 (15)	
H5	0.5660	0.8706	0.3150	0.057*	
C6	0.5266 (9)	0.9218 (8)	0.1915 (4)	0.0432 (14)	
H6	0.6604	0.9397	0.1802	0.052*	
C7	0.2060 (8)	0.8066 (7)	0.3769 (4)	0.0395 (13)	
C8	0.0424 (9)	0.7481 (8)	0.5080 (4)	0.0418 (13)	
C9	−0.0929 (8)	0.7148 (7)	0.5800 (4)	0.0379 (13)	
C10	−0.2927 (9)	0.7063 (8)	0.5682 (4)	0.0442 (14)	
H10	−0.3440	0.7292	0.5160	0.053*	
C11	−0.4144 (9)	0.6632 (8)	0.6357 (4)	0.0495 (15)	
H11	−0.5479	0.6593	0.6276	0.059*	
C12	−0.1584 (10)	0.6393 (9)	0.7221 (4)	0.0559 (17)	
H12	−0.1112	0.6178	0.7757	0.067*	
C13	−0.0248 (9)	0.6813 (8)	0.6596 (4)	0.0497 (15)	
H13	0.1090	0.6871	0.6705	0.060*	

### Atomic displacement parameters (Å^2^)

**Table:**

	*U*^11^	*U*^22^	*U*^33^	*U*^12^	*U*^13^	*U*^23^
Cu1	0.0421 (6)	0.0432 (6)	0.0477 (6)	0.0197 (5)	0.0129 (4)	0.0186 (5)
S1	0.0490 (9)	0.0680 (11)	0.0412 (9)	0.0289 (8)	0.0147 (7)	0.0281 (8)
S2	0.0484 (9)	0.0420 (9)	0.0663 (11)	0.0258 (7)	0.0192 (8)	0.0252 (8)
N1	0.044 (3)	0.043 (3)	0.052 (3)	0.026 (2)	0.015 (2)	0.020 (2)
N2	0.032 (2)	0.037 (3)	0.038 (3)	0.014 (2)	0.009 (2)	0.015 (2)
N3	0.045 (3)	0.063 (3)	0.043 (3)	0.022 (3)	0.012 (2)	0.023 (3)
N4	0.048 (3)	0.064 (3)	0.041 (3)	0.021 (3)	0.014 (2)	0.027 (3)
N5	0.050 (3)	0.064 (4)	0.044 (3)	0.022 (3)	0.017 (2)	0.022 (3)
O1	0.035 (2)	0.043 (2)	0.051 (2)	0.0140 (17)	0.0117 (17)	0.0118 (18)
O2	0.055 (3)	0.059 (3)	0.051 (3)	0.013 (2)	0.018 (2)	0.011 (2)
C1	0.031 (3)	0.045 (3)	0.031 (3)	0.014 (2)	0.011 (2)	0.016 (3)
C2	0.041 (3)	0.052 (4)	0.043 (3)	0.018 (3)	0.007 (3)	0.023 (3)
C3	0.035 (3)	0.056 (4)	0.046 (3)	0.013 (3)	0.009 (3)	0.022 (3)
C4	0.040 (3)	0.034 (3)	0.036 (3)	0.011 (2)	0.012 (2)	0.009 (2)
C5	0.042 (3)	0.064 (4)	0.045 (4)	0.026 (3)	0.009 (3)	0.025 (3)
C6	0.040 (3)	0.054 (4)	0.043 (3)	0.022 (3)	0.017 (3)	0.020 (3)
C7	0.042 (3)	0.039 (3)	0.036 (3)	0.014 (3)	0.009 (3)	0.012 (3)
C8	0.047 (3)	0.043 (3)	0.037 (3)	0.017 (3)	0.006 (3)	0.014 (3)
C9	0.043 (3)	0.035 (3)	0.036 (3)	0.015 (2)	0.008 (2)	0.012 (3)
C10	0.046 (3)	0.053 (4)	0.047 (3)	0.026 (3)	0.009 (3)	0.026 (3)
C11	0.041 (3)	0.052 (4)	0.058 (4)	0.020 (3)	0.012 (3)	0.017 (3)
C12	0.054 (4)	0.075 (5)	0.042 (4)	0.023 (3)	0.014 (3)	0.026 (3)
C13	0.043 (3)	0.066 (4)	0.043 (4)	0.022 (3)	0.007 (3)	0.023 (3)

### Geometric parameters (Å, °)

**Table:**

Cu1—N1	2.071 (4)	O2—H2C	0.8159
Cu1—N1^i^	2.071 (4)	C2—C3	1.372 (8)
Cu1—O1^i^	2.118 (4)	C2—H2	0.9300
Cu1—O1	2.118 (4)	C3—C4	1.365 (8)
Cu1—N2	2.178 (4)	C3—H3	0.9300
Cu1—N2^i^	2.178 (4)	C4—C5	1.388 (8)
S1—C7	1.725 (6)	C4—C7	1.485 (7)
S1—C8	1.726 (5)	C5—C6	1.372 (7)
S2—C1	1.638 (6)	C5—H5	0.9300
N1—C1	1.150 (7)	C6—H6	0.9300
N2—C6	1.324 (7)	C8—C9	1.479 (7)
N2—C2	1.325 (7)	C9—C13	1.382 (7)
N3—C7	1.301 (7)	C9—C10	1.384 (7)
N3—N4	1.375 (6)	C10—C11	1.382 (8)
N4—C8	1.300 (7)	C10—H10	0.9300
N5—C11	1.305 (7)	C11—H11	0.9300
N5—C12	1.342 (8)	C12—C13	1.372 (8)
O1—H1B	0.8214	C12—H12	0.9300
O1—H1C	0.8200	C13—H13	0.9300
O2—H2B	0.8172		
			
N1—Cu1—N1^i^	180.000 (1)	C4—C3—H3	120.5
N1—Cu1—O1^i^	89.03 (16)	C2—C3—H3	120.5
N1^i^—Cu1—O1^i^	90.97 (16)	C3—C4—C5	118.0 (5)
N1—Cu1—O1	90.97 (16)	C3—C4—C7	121.8 (5)
N1^i^—Cu1—O1	89.03 (17)	C5—C4—C7	120.2 (5)
O1^i^—Cu1—O1	180.0	C6—C5—C4	118.4 (5)
N1—Cu1—N2	91.15 (17)	C6—C5—H5	120.8
N1^i^—Cu1—N2	88.85 (17)	C4—C5—H5	120.8
O1^i^—Cu1—N2	86.47 (15)	N2—C6—C5	124.1 (5)
O1—Cu1—N2	93.53 (15)	N2—C6—H6	118.0
N1—Cu1—N2^i^	88.85 (17)	C5—C6—H6	118.0
N1^i^—Cu1—N2^i^	91.15 (17)	N3—C7—C4	124.0 (5)
O1^i^—Cu1—N2^i^	93.53 (15)	N3—C7—S1	113.8 (4)
O1—Cu1—N2^i^	86.47 (14)	C4—C7—S1	122.1 (4)
N2—Cu1—N2^i^	180.0 (2)	N4—C8—C9	123.7 (5)
C7—S1—C8	87.1 (3)	N4—C8—S1	113.7 (4)
C1—N1—Cu1	159.4 (4)	C9—C8—S1	122.6 (4)
C6—N2—C2	116.5 (5)	C13—C9—C10	118.0 (5)
C6—N2—Cu1	121.5 (4)	C13—C9—C8	119.9 (5)
C2—N2—Cu1	122.0 (3)	C10—C9—C8	122.0 (5)
C7—N3—N4	112.5 (5)	C11—C10—C9	118.6 (5)
C8—N4—N3	112.8 (4)	C11—C10—H10	120.7
C11—N5—C12	117.1 (5)	C9—C10—H10	120.7
Cu1—O1—H1B	118.7	N5—C11—C10	124.0 (6)
Cu1—O1—H1C	124.5	N5—C11—H11	118.0
H1B—O1—H1C	108.3	C10—C11—H11	118.0
H2B—O2—H2C	110.6	N5—C12—C13	123.5 (6)
N1—C1—S2	179.8 (5)	N5—C12—H12	118.2
N2—C2—C3	123.9 (5)	C13—C12—H12	118.2
N2—C2—H2	118.0	C12—C13—C9	118.7 (6)
C3—C2—H2	118.0	C12—C13—H13	120.6
C4—C3—C2	119.0 (5)	C9—C13—H13	120.6

Symmetry codes: (i) −*x*+1, −*y*+2, −*z*.

### Hydrogen-bond geometry (Å, °)

**Table:**

*D*—H···*A*	*D*—H	H···*A*	*D*···*A*	*D*—H···*A*
O2—H2B···N5	0.82	2.03	2.835 (6)	169
O2—H2C···S2^ii^	0.82	2.90	3.541 (4)	137
O1—H1B···S2^iii^	0.82	2.50	3.303 (4)	164
O1—H1C···O2^iv^	0.82	1.95	2.761 (6)	171

Symmetry codes: (ii) −*x*, −*y*+1, −*z*+1; (iii) *x*−1, *y*, *z*; (iv) *x*+1, *y*, *z*−1.
